# The Ninth International Medical Congress

**Published:** 1886-01

**Authors:** 


IN the December number of the JOURNAL AND EXAMINER a detailed account was given of preliminaries to the organization of *The Ninth International Medical Congress*, ending with the election, by the *Executive Committee* of the congress, of six additional members as authorized by the rules adopted for the government of the congress.

It appears that after the six newly-elected members had been officially notified of their election, a meeting was called, in Philadelphia, to confer as to the advisability of all or any of the newly-elected members accepting the positions to which they had been elected. The result of that meeting has been announced, in various journals of the country, in the following terms :

THE INTERNATIONAL MEDICAL CONGRESS OF 1887.—A meeting of members of the medical profession interested in the International Medical Congress in 1887, to which prominent medical men from a number of cities were invited, was held at the hall of the College of Physicians, Philadelphia, December 4, 1885, Dr. D. Hayes Agnew in the chair.

It was stated that official notice had been given of the election, as members of the present Executive Committee of the congress, of Dr. J. S. Billings, United States army, and Dr. J.

M. Browne, United States navy; Dr. Christopher Johnston, of Baltimore; Dr. George J. Englemann, of St. Louis; and Dr. J. M. Da Costa and Dr. William Pepper, of Philadelphia.

A general and strong expression of opinion was made in support of the American Medical Association and its Code of Medical Ethics, and sincere regret was expressed that hasty action on the part of the association, and the introduction of false issues, had imperilled the success of the congress. It was made entirely evident, however, that the acceptance of the above elections would not be regarded as affording any adequate guarantee for the future scientific conduct of the congress, and, consequently, would not be followed by any co-operation on the part of the leading members of the profession now unwilling to join in that work. As an evidence of the earnest desire which is felt for the restoration of harmony upon this subject, and for the reorganization of the congress on a basis which would command general support, and thus insure success, the view was unanimously expressed that if the present Executive Committee should unite with them the Original Enlarged General Committee, and recommence the organization *de novo*, this course would ensure the desired result.

The *Executive Committee* of the congress has seemed to manifest a desire to have the congress organized as a truly representative body, and has taken such steps to secure the co-operation of many prominent members of the medical profession in the United States who are not in the preliminary organization of the congress, as could properly be done, under the rules adopted by the Committee of Arrangements, before it transferred the affairs of the congress to the *Executive Committee*, and that *Committee* accepted the duties of that office subject to those rules. Of these Rule 10 limits the number of the *Executive Committee* to thirty members.

As the committee which presented the invitation at Copenhagen numbered eight, and to this was added the "General

Committee," which numbered twenty-eight, making thirty-six, that number alone would exceed by six members the number authorized as the limit of the *Executive Committee*, exclusive of any members of the Executive Committee. It appears that since that meeting in Philadelphia all six of the newly-elected members declined to accept the positions to which they had been elected, and it is understood that in so doing at least some of the six expressed a willingness to accept if the conditions proposed at the Philadelphia meeting should be accepted by the *Executive Committee*, conditions which, it would seem, are impracticable, if the rules adopted for the government of the congress are to be enforced, in which case it must be inferred that those six gentlemen and their friends, whom the *Executive Committee* appears to have desired to have co-operate, decline to do so, except on conditions which it is not in the power of the *Executive Committee* to accept, because of the existing rules, adopted before the *Executive Committee* came into existence, and which the *Executive Committee* has no authority to change.

It would seem as if that *Committee* had shown a just comprehension of its duties, in the trust confided to it, in electing those six gentlemen to official positions, and in leaving vacant the presidencies of three important sections, and, in addition, nearly all of the other most important offices in the various sections of the congress, which were not already filled, when the Executive Committee was appointed, by the *Committee of Arrangements*. It thus gave to those six gentlemen an opportunity to participate in filling those positions with such officers as might be deemed best qualified for them. It may be said that there was no necessity on the part of the Executive Committee of tendering these positions to those six gentlemen; but evidently the Committee considered that it was the right thing for it to do, and having done it there are those who inquire what could have been the motive which prompted them to decline to accept their election, except upon a condition which they must have known would be in direct conflict with the rules already adopted for the government of the congress, according to the precedent set by each preceding congress.

Would it not have been better for the gentlemen themselves, and for the cause of humanity and of science, that personal preferences should have been subordinated, and that they should have met the Executive Committee in the same spirit in which the tender was made, in order to contribute to the success of so important an occasion, where good faith, professional pride and patriotism combine to make it the duty of the whole profession of our country to unite in a determined effort to make the next congress what it ought to be?

Since the well-known character of those six gentlemen precludes any other idea than that they earnestly desire that the congress shall be an eminent success, from every point of view, let us hope that further consideration will result in such modification of their action that they will not impose impracticable conditions when the *Executive Committee* shows a disposition to go as far to meet their wishes as its sense of duty and of honor will permit, in acting under the trust it has accepted.

